# Intra-Articular Leukocyte-Poor Platelet-Rich Plasma Injections for Japanese Patients With Osteoarthritis of the Knee: A Three-Year Observational Retrospective Study After Phase 1 and Phase 2a Trials

**DOI:** 10.7759/cureus.30490

**Published:** 2022-10-19

**Authors:** Yu Taniguchi, Tomokazu Yoshioka, Hisashi Sugaya, Katsuya Aoto, Akihiro Kanamori, Masashi Yamazaki

**Affiliations:** 1 Orthopaedic Surgery, Ichihara Hospital, Tsukuba, JPN; 2 Orthopaedic Surgery, Faculty of Medicine, University of Tsukuba, Tsukuba, JPN; 3 Orthopaedic Surgery, Faculty of Medicine, Tsukuba University of Technology, Tsukuba, JPN

**Keywords:** leukocyte-poor prp, japanese population, mid-term results, intra-articular injection, platelet-rich plasma, osteoarthritis

## Abstract

Background: We have previously confirmed the safety and feasibility of intra-articular (IA) platelet-rich plasma (PRP) injections in Japanese patients with osteoarthritis (OA) of the knee. This study aimed to investigate the clinical and radiological outcomes in patients who were followed up for three years.

Methods: Nine patients were evaluated in this observational study. All the patients were women with a mean age of 60.6 years. PRP was prepared by single centrifugation and classified as leukocyte-poor (LP)-PRP, and was administered via three IA injections at weekly intervals. Patients were evaluated at baseline and final follow-up using the Kellgren-Lawrence (KL) grade and the Japanese Orthopedics Association (JOA) score. The need for additional treatment was also investigated at the final follow-up.

Results: At a mean follow-up of three years, the JOA score improved from 75 points (p) to 83.8 p. The sub-categorical scores changed as follows: gait, 23.1-26.9 p; stairs, 12.5-17.5 p; range of motion, 30-30 p; and swelling, 9.4-10 p. While the KL grade was maintained in six patients, it progressed in two patients from I to II and II to III, respectively. Two patients received additional treatment at the final follow-up.

Conclusions: At the final follow-up, the functional knee score, especially gait and the ability to go up and down the stairs, improved in six out of eight patients without additional treatment. These results suggest that LP-PRP injections produced safe outcomes without OA-worsening in most patients at three-year follow-up.

## Introduction

This article was previously presented as a meeting abstract at the 12th ISAKOS Biennial Congress 2019 on May 12, 2019. Knee osteoarthritis (KOA) is a condition caused by regression and degeneration of the joint, which affects many middle-aged and elderly individuals. It is one of the most common chronic degenerative joint diseases affecting patients’ quality of life [1]. In 2009, radiographic KOA was estimated in 25.3 million individuals aged ≥40 years, while symptomatic KOA was present in approximately 7.8 million individuals in Japan [2], and these numbers are expected to increase. Although knee replacement surgery provides an effective solution for severe KOA [3], the main treatments for painful KOA in the early to middle stages are conservative nonsurgical interventions [4]. These include exercise, weight loss, physical therapy, and pharmacological options, such as non-steroidal anti-inflammatory medication, oral opioids, and injection therapy [5-6].

Platelet-rich plasma (PRP) injection is a nonsurgical intervention for the treatment of KOA. It is obtained by centrifuging autologous peripheral blood, providing a high concentration of platelets in a small volume of plasma. Activation of PRP releases an initial burst, followed by a sustained release of biologically active growth factors and other molecules, including platelet-derived growth factor, transforming growth factor-β, type I insulin-like growth factor, and vascular endothelial growth factor [7]. The rationale for using PRP injections for KOA treatment is based on the modulation of the intra-articular (IA) environment by introducing autologous blood products in the joint, reducing inflammatory distress, and promoting chondrogenesis [8-10]. Recent systematic reviews comparing hyaluronic acid (HA) and saline with PRP found that the latter provided more pain relief and produced better functional outcomes in patients with KOA at one year after the injection, without an increase in the risk of adverse events [8, 11]. Thus, although several systematic reviews have shown good short-term results (usually 6-12 months after the injection), a longer-term evaluation could reveal differences in the benefits of clinical outcomes. We have previously confirmed the safety and feasibility of IA PRP injections in Japanese patients with KOA [12]. This study aimed to investigate the clinical and radiological outcomes in patients who were followed up for three years.

## Materials and methods

The Phase 1 and Phase 2a clinical trials were approved by the appropriate Institutional Review Board (Application no. H25-040) of the University of Tsukuba Hospital, as published previously in a six-month study [12]. In the previous clinical trial, inclusion criteria were an age of 50-75 years; history of chronic (at least 3 months) knee joint pain, defined as a visual analog scale score of >35 mm (on a 0-100 mm scale); radiographically documented KOA grade I-III according to the Kellgren-Lawrence (KL) radiographic classification scale; and a body mass index of 20-32 kg/m2. The study sample reflected the individuals with an average Japanese physique who required treatment of one knee only. The exclusion criteria were polyarticular disease; knee arthroscopy within the previous year; HA or steroid IA infiltration within the previous 3 months; history of infectious disease; systemic disorders, including diabetes and rheumatoid arthritis; hematological diseases (coagulopathy); severe cardiovascular diseases; immunodepression; therapy with anticoagulants or anti-aggregating agents; use of non-steroidal anti-inflammatory drugs two weeks before blood sampling; and hemoglobin levels <10 g/dL.

Initially, the study included 10 patients who were administered IA PRP injections three times at weekly intervals. All the participants were women, with a mean age of 60.6 years (range, 51-70 years). Based on the KL radiographic classification, four patients were classified as having grade I, four with grade II, and two with grade III KOA. PRP was prepared by single centrifugation (2100 rpm, 8 min, PRGF-Endoret, BTI Biotechnology Institute, Vitoria, Spain) and classified as leukocyte-poor (LP)-PRP. The findings of the hematological analysis and growth factor concentrations have been presented previously [12]. The treatment involved three IA injections of 6.0 mL each at weekly intervals. After each injection, the patients were instructed to refrain from physical exercise for at least 24 h, although no other restrictions were specified regarding activities of daily living. All patients visited the outpatient clinic once prior to treatment, at one, three, and six months after the treatment, and every three months thereafter for three years. Patients were evaluated at baseline and at the final follow-up using the Japanese Orthopedics Association (JOA) score, a standard tool for assessing knee function in Japan (Table 1). Radiographs in the supine anteroposterior, lateral, and skyline views were obtained during the same visit. Two authors (YT and TY) reviewed all the radiographs and graded them using the KL radiographic classification scale [13]. The joint space width (JSW) at the narrowest point (minimum JSW [mJSW]) in the medial or lateral compartment of the femorotibial joints and the total osteophyte areas on the proximal tibia and distal femur were calculated from the calibrated radiographs using the KOA computer-aided diagnosis (KOACAD) system (INOTECH Corporation, Hiroshima, Japan) [14]. Patients were assessed for further treatment requirements at their final follow-up.

**Table 1:** Criteria for evaluation of osteoarthritis of the knee according to the Japanese Orthopaedic Association [15].

Domain	Points	
Pain on walking	Right	Left
Ability to walk 1 km or more usually without pain, or only occasionally mild pain	30	30
Ability to walk 1 km or more regardless of pain	25	25
Ability to walk 500 m or more, but less than 1 km	20	20
Ability to walk 100 m or more, but less than 500 m	15	15
Ability to walk indoors or nearby, but less than 100 m	10	10
Inability to walk	5	5
Inability to stand	0	0
Pain on ascending or descending stairs		
No pain	25	25
Pain relieved by using handrails	20	20
Pain with handrails, but no pain with step-by-step ambulation	15	15
Pain with step-by-step ambulation: relieved by using handrails	10	10
Pain even with step-by-step ambulation and handrail use	5	5
Inability to ascend or descend due to pain	0	0
Range of movement		
Ability to squat	35	35
Ability to sit sideways or cross-legged sitting	30	30
Flexion or arc of movement of 110° or more	25	25
Flexion or arc of movement of 75° or more	20	20
Flexion or arc of movement of 35° or more	10	10
Flexion or arc of movement less than 35° including ankylosis or severe flexion contracture	0	0
Joint effusion		
No effusion, no swelling	10	10
Occasional aspiration required	5	5
Frequent aspiration required	0	0
Total score	100	100

Statistical analysis

Differences between the pre-treatment and final follow-up measurements were evaluated using the Student’s t-test. Statistical significance was set at a p-value of <0.05. All statistical analyses were performed using SPSS Statistics 21.0 (International Business Machines Co., New York, NY, USA).

## Results

**Figure 1:**
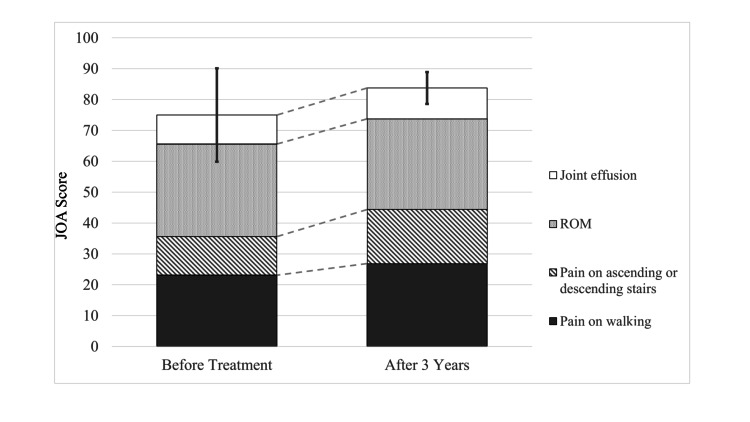
Japanese Orthopedic Association scores before and after treatment. Different filling patterns represent different categories. ROM, range of motion

**Figure 2:**
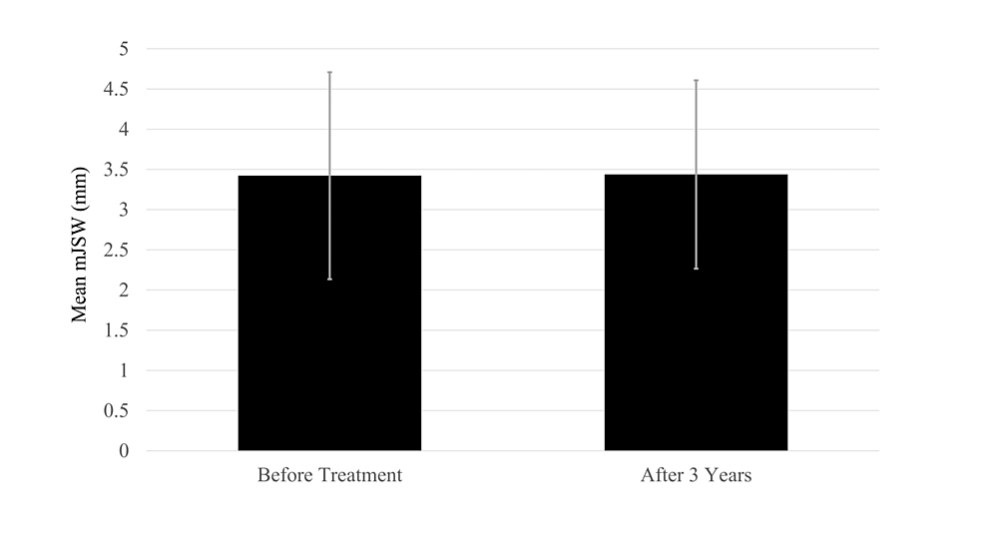
Mean mJSW. mJSW, minimum joint space width

Patient demographics are summarized in Table 2. Excluding one patient who could not be followed up, nine knees of nine patients were reviewed retrospectively (the follow-up rate was 90%). No serious adverse events occurred during the follow-up period. The mean age of patients receiving IA PRP was 64.3 years (range, 51-70 years), and the mean follow-up period was 36 ± 11.1 months (range, 7-47 months). One patient with KL grade III KOA with a varus deformity and anterior cruciate ligament (ACL) mucoid degeneration underwent total knee arthroplasty seven months after the PRP injections. The JOA score of the remaining eight patients improved from 75 points (p) to 83.8 p. Changes in sub-categorical scores were gait: 23.1-26.9 p, stairs: 12.5-17.5 p, range of motion: 30-30 p, and swelling: 9.4-10 p (Figure 1). Mean JOA scores were assessed at baseline (before injection) and the final follow-up visit. Radiographic findings revealed that while the KL grade was maintained in six patients (75%), it progressed in two patients from grade I to grade II and grade II to grade III, respectively. The mean mJSW was assessed by the KOACAD system at baseline and at the final follow-up visit. The mean mJSW before the IA PRP injections and at the final follow-up visit were 3.42 ± 1.29 and 3.44 ± 1.17 mm, respectively, and there was no statistically significant difference between the scores (Figure 2). At the final follow-up, two patients (25%) had received additional treatments; one had a lateral wedge insole and the other received HA injections.

**Table 2:** Demographic and clinical characteristics of 10 patients in Phase 1 and Phase 2a clinical trial. yr, year; F, female; Y, yes; N, no; mo, month; IA-PRP, intra-articular-platelet rich plasma; KL, Kellgren-Lawrence radiographic classification scale; TKA, total knee arthroplasty; HA, hyaluronic acid

Patient	Age (yr)/sex	Follow-up	Follow-up period (mo)	KL grade before IA-PRP	KL grade at the final follow-up	Additional treatment at the final follow-up
1	59/F	N	-	3	-	-
2	65/F	Y	47	2	2	
3	58/F	Y	45	3	3	
4	74/F	Y	43	1	2	Lateral wedge insole
5	56/F	Y	39	1	1	
6	68/F	Y	38	3	3	
7	74/F	Y	36	2	3	
8	60/F	Y	35	2	2	
9	70/F	Y	7	3	TKA	TKA
10	54/F	Y	33	2	2	HA injection

## Discussion

The most important finding of this study was that the functional knee scores, especially those related to gait and the ability to climb up the stairs, improved in six out of eight (75%) patients without any additional treatment at the final follow-up. These results suggest that LP-PRP injections produce safe outcomes without KOA worsening in most patients at three-year follow-up. The complex pathogenesis of KOA involves mechanical, inflammatory, and metabolic factors, ultimately leading to structural destruction and failure of the synovial joint. The disease involves dynamic changes resulting from an imbalance between the destruction and repair of the joint tissue [5, 16]. The PRP contains growth factors that stimulate cellular anabolism, inflammatory mediators, and modulators that exert anti-inflammatory effects. Upon PRP administration to an unbalanced KOA, the action of these growth factors balances repair and destruction, thus promoting the restoration of joint homeostasis [17]. Multiple systematic reviews have shown that PRP is clinically superior to HA and saline for the relief of symptomatic knee pain [8, 11, 18-19]. However, since these reviews cover trials with a follow-up period of only 12 months, the long-term effects of PRP remain unclear, and few reports have discussed them. Di Martino et al. compared the long-term outcomes of leukocyte-rich PRP and HA injections (three per week) with a mean follow-up of 64.3 months [20] and found no significant differences in either the clinical outcomes or effect duration. However, there was a significant difference in the reinjection rate at 24 months and the PRP group had a lower reinjection rate at the five-year evaluation. Kon et al. reported that IA injections of autologous protein solution for mild to moderate KOA are safe, resulting in a significant decrease in pain three years after a single injection [21]. The present study shows the JOA score, especially for gait and the ability to go up and down stairs, improved in six of the eight patients (75%) without additional treatment at a mean three-year follow-up. The JOA score is a reliable and valid tool for evaluating the functional status of patients with KOA [15] and is frequently used for knee scoring in Japanese clinical practice. Therefore, LP-PRP injections may reduce the likelihood of disease progression over a three-year period in patients with mild and moderate KOA.

 In the present study, knee radiographs were obtained for evaluation before the intervention and at the final follow-up. Progression of the KL grade was observed in two of the eight cases. Yoshimura et al. followed up untreated KOA patients for three years and reported KL grade progression at an annual rate of 8% [22]. Radiographic findings in this study showed results equivalent to those of the group without intervention. Therefore, the PRP injection may not be sufficiently effective to prevent the deterioration of radiographic KOA. Evaluation using the KOACAD system showed no significant difference in the mJSW before treatment and at the final follow-up. The KOACAD system is fully automated for quantifying the major features of KOA on standard radiographs, allowing for an objective, accurate, and straightforward assessment of the structural severity of KOA in general clinical practice [13, 23-24]. Mazzuca et al. reported that the mean annual joint space narrowing (JSN) ranged from 0.06 mm to 0.60 mm [25]. Pavelka et al. reported that the JSN progressed by 0.40 mm over two years in patients diagnosed with KOA [26]. The mJSW values in the present study using the KOACAD system for three years were superior to those reported in the earlier studies [25-26]. However, it is possible that knee radiographs were obtained in the non-weight-bearing position and the JSN was not accurately evaluated. Different results may have been obtained if the knee radiographs were obtained from a weight-bearing position.

The present study has several limitations. Firstly, it is based on a relatively small sample size, as only eight patients were followed up for three years. Although the clinical scores improved compared to those before the intervention, no statistically significant differences were observed, given the small sample size. Secondly, this was an open-label study, with no control group for comparison. As previously reported and widely documented for knee injection studies [27-28], patients receiving regenerative therapy are susceptible to the placebo effect, which cannot be ruled out in this single-arm PRP study. Thirdly, we used simple X-rays and not MRI for image evaluation. MRI can be used to evaluate cartilage, meniscus, and bone marrow lesion in the knee joint and is better for observing changes in KOA over time [29-30].

## Conclusions

The use of IA LP PRP injections for mild to moderate KOA was safe and resulted in no aggravation of pain for three years after the treatment (three injections weekly). Although further research is needed to confirm the true efficacy of this treatment, these results suggest that the therapeutic effects of LP-PRP injections may have mid-term durability.

## References

[1] Cross M, Smith E, Hoy D (2014). The global burden of hip and knee osteoarthritis: estimates from the global burden of disease 2010 study. Ann Rheum Dis.

[2] Yoshimura N, Muraki S, Oka H (2009). Prevalence of knee osteoarthritis, lumbar spondylosis, and osteoporosis in Japanese men and women: the research on osteoarthritis/osteoporosis against disability study. J Bone Miner Metab.

[3] McAlindon TE, LaValley MP, Harvey WF, Price LL, Driban JB, Zhang M, Ward RJ (2017). Effect of intra-articular triamcinolone vs saline on knee cartilage volume and pain in patients with knee osteoarthritis: a randomized clinical trial. JAMA.

[4] Cook CS, Smith PA (2018). Clinical update: why PRP should be your first choice for injection therapy in treating osteoarthritis of the knee. Curr Rev Musculoskelet Med.

[5] Hunter DJ, Bierma-Zeinstra S (2019). Osteoarthritis. Lancet.

[6] Sharma L (2021). Osteoarthritis of the knee. N Engl J Med.

[7] Taniguchi Y, Yoshioka T, Sugaya H, Gosho M, Aoto K, Kanamori A, Yamazaki M (2019). Growth factor levels in leukocyte-poor platelet-rich plasma and correlations with donor age, gender, and platelets in the Japanese population. J Exp Orthop.

[8] Dai WL, Zhou AG, Zhang H, Zhang J (2017). Efficacy of platelet-rich plasma in the treatment of knee osteoarthritis: a meta-analysis of randomized controlled trials. Arthroscopy.

[9] Bennell KL, Hunter DJ, Paterson KL (2017). Platelet-rich plasma for the management of hip and knee osteoarthritis. Curr Rheumatol Rep.

[10] Filardo G, Di Matteo B, Di Martino A (2015). Platelet-rich plasma intra-articular knee injections show no superiority versus viscosupplementation: a randomized controlled trial. Am J Sports Med.

[11] Hohmann E, Tetsworth K, Glatt V (2020). Is platelet-rich plasma effective for the treatment of knee osteoarthritis? A systematic review and meta-analysis of level 1 and 2 randomized controlled trials. Eur J Orthop Surg Traumatol.

[12] Taniguchi Y, Yoshioka T, Kanamori A, Aoto K, Sugaya H, Yamazaki M (2018). Intra-articular platelet-rich plasma (PRP) injections for treating knee pain associated with osteoarthritis of the knee in the Japanese population: a phase I and IIa clinical trial. Nagoya J Med Sci.

[13] Kellgren JH, Lawrence JS (1957). Radiological assessment of osteo-arthrosis. Ann Rheum Dis.

[14] Oka H, Muraki S, Akune T (2008). Fully automatic quantification of knee osteoarthritis severity on plain radiographs. Osteoarthritis Cartilage.

[15] Okuda M, Omokawa S, Okahashi K, Akahane M, Tanaka Y (2012). Validity and reliability of the Japanese Orthopaedic Association score for osteoarthritic knees. J Orthop Sci.

[16] Fu K, Robbins SR, McDougall JJ (2018). Osteoarthritis: the genesis of pain. Rheumatology (Oxford).

[17] Xie X, Zhang C, Tuan RS (2014). Biology of platelet-rich plasma and its clinical application in cartilage repair. Arthritis Res Ther.

[18] Belk JW, Kraeutler MJ, Houck DA, Goodrich JA, Dragoo JL, McCarty EC (2021). Platelet-rich plasma versus hyaluronic acid for knee osteoarthritis: a systematic review and meta-analysis of randomized controlled trials. Am J Sports Med.

[19] Meheux CJ, McCulloch PC, Lintner DM, Varner KE, Harris JD (2016). Efficacy of intra-articular platelet-rich plasma injections in knee osteoarthritis: a systematic review. Arthroscopy.

[20] Di Martino A, Di Matteo B, Papio T (2019). Platelet-rich plasma versus hyaluronic acid injections for the treatment of knee osteoarthritis: results at 5 years of a double-blind, randomized controlled trial. Am J Sports Med.

[21] Kon E, Engebretsen L, Verdonk P, Nehrer S, Filardo G (2020). Autologous protein solution injections for the treatment of knee osteoarthritis: 3-year results. Am J Sports Med.

[22] Yoshimura N, Muraki S, Oka H, Tanaka S, Kawaguchi H, Nakamura K, Akune T (2012). Accumulation of metabolic risk factors such as overweight, hypertension, dyslipidaemia, and impaired glucose tolerance raises the risk of occurrence and progression of knee osteoarthritis: a 3-year follow-up of the ROAD study. Osteoarthritis Cartilage.

[23] Oka H, Muraki S, Akune T, Nakamura K, Kawaguchi H, Yoshimura N (2010). Normal and threshold values of radiographic parameters for knee osteoarthritis using a computer-assisted measuring system (KOACAD): the ROAD study. J Orthop Sci.

[24] Sasaki E, Tsuda E, Yamamoto Y (2015). Serum hyaluronic acid concentration predicts the progression of joint space narrowing in normal knees and established knee osteoarthritis - a five-year prospective cohort study. Arthritis Res Ther.

[25] Mazzuca SA, Brandt KD, Katz BP (1997). Is conventional radiography suitable for evaluation of a disease-modifying drug in patients with knee osteoarthritis?. Osteoarthr Cartil.

[26] Pavelka K, Forejtová S, Olejárová M (2004). Hyaluronic acid levels may have predictive value for the progression of knee osteoarthritis. Osteoarthritis Cartilage.

[27] Previtali D, Merli G, Di Laura Frattura G, Candrian C, Zaffagnini S, Filardo G (2021). The long-lasting effects of "Placebo Injections" in knee osteoarthritis: a meta-analysis. Cartilage.

[28] Kon E, Engebretsen L, Verdonk P, Nehrer S, Filardo G (2018). Clinical outcomes of knee osteoarthritis treated with an autologous protein solution injection: a 1-year pilot double-blinded randomized controlled trial. Am J Sports Med.

[29] Dório M, Hunter DJ, Collins JE (2020). Association of baseline and change in tibial and femoral cartilage thickness and development of widespread full-thickness cartilage loss in knee osteoarthritis - data from the Osteoarthritis Initiative. Osteoarthritis Cartilage.

[30] Runhaar J, Schiphof D, van Meer B, Reijman M, Bierma-Zeinstra SM, Oei EH (2014). How to define subregional osteoarthritis progression using semi-quantitative MRI osteoarthritis knee score (MOAKS). Osteoarthritis Cartilage.

